# Postbiotics in oncology: science or science fiction?

**DOI:** 10.3389/fmicb.2023.1182547

**Published:** 2023-08-07

**Authors:** Anna Kudra, Karolina Kaźmierczak-Siedlecka, Bartosz Kamil Sobocki, Damian Muszyński, Joanna Połom, Ludovico Carbone, Luigi Marano, Franco Roviello, Leszek Kalinowski, Ewa Stachowska

**Affiliations:** ^1^Scientific Circle of Studies Regarding Personalized Medicine Associated With Department of Medical Laboratory Diagnostics—Fahrenheit Biobank BBMRI.pl, Medical University of Gdańsk, Gdańsk, Poland; ^2^Department of Medical Laboratory Diagnostics—Fahrenheit Biobank BBMRI.pl, Medical University of Gdańsk, Gdańsk, Poland; ^3^Scientific Circle of Oncology and Radiotherapy, Medical University of Gdańsk, Gdańsk, Poland; ^4^Department of Medicine Surgery and Neuroscience, University of Siena, Siena, Italy; ^5^BioTechMed Centre/Department of Mechanics of Materials and Structures, Gdańsk University of Technology, Gdańsk, Poland; ^6^Department of Human Nutrition and Metabolomics, Pomeranian Medical University, Szczecin, Poland

**Keywords:** gut microbiome, cancer, postbiotics, exopolysaccharides, tryptophan metabolites, short chain fatty acids, cell wall fragments, extracellular vesicles

## Abstract

The gut microbiome has been increasingly understood to play a critical role in carcinogenesis and cancer disease progression. The most recent research advancements have shown that different tools of microbiota manipulation contribute to gut microbiome–immune–oncology axis modulation, offering exciting opportunities for targeted interventions aimed at improving the efficacy of established anti-cancer therapy. Postbiotics are a new entry among the biotics showing beneficial effects on human health while not requiring living cells to obtain the health effect and therefore not subjected to food safety rules for live microorganisms. Postbiotics are recently defined as the “preparation of inanimate microorganisms and/or their components that confers a health benefit on the host” and have gradually become the focus of the scientific community. Since the beginning of research on this topic, numerous studies about postbiotics have been proven to strengthen the gut barrier, reduce inflammation, and promote antimicrobial activity. However, research on the potential application of cancer therapy is still at the early stages of its efforts to uncover all the secrets surrounding postbiotics. This review aims to increase our understanding of the anti-cancer effect of postbiotics throughout a “bibliographic journey” on the biological activity of their components, including exopolysaccharides, cell wall fragments, tryptophan metabolites, enzymes, bacterial lysates, extracellular vesicles, and short-chain fatty acids, highlighting their perspective as a new supportive therapeutic method of treatment and identifying the literature gaps where further research is needed.

## 1. Introduction

**Figure 1:**
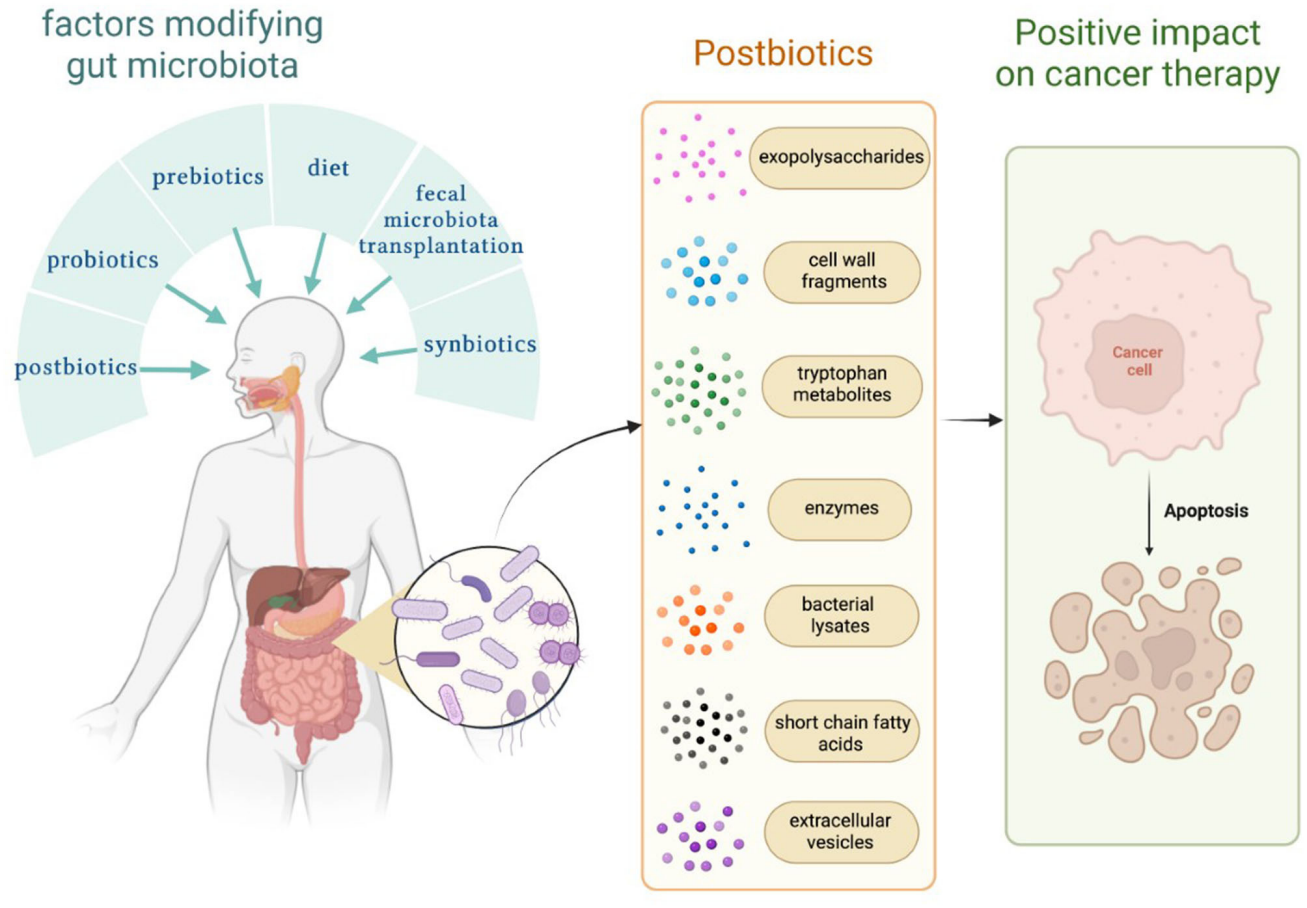
Factors that modify the gut microbiome and substances known as postbiotics. Own elaboration based on the literature (Tsilingiri and Rescigno, 2013; Kazmierczak-Siedlecka et al., 2020; Malagón-Rojas et al., 2020). This figure was created using Biorender.com.

The gut microbiome plays a crucial role in the human body. The balance of bacterial, fungal, and viral parts of the gut microbiota allows for maintaining gut homeostasis. There are several methods, which modify gut microbiota, such as diet, probiotics, next-generation probiotics, synbiotics, fecal microbiota transplantation, different treatment modalities, and postbiotics (Kazmierczak-Siedlecka et al., 2020, 2022b). Nowadays, the definition of postbiotics has not been precisely described; however, a few assessments were addressed. Postbiotics are defined as bioactive compounds which are produced during a fermentation process (Malagón-Rojas et al., 2020). Postbiotics regard substances that are produced through the metabolic activity of microbes, and they provide a beneficial effect to the host both indirectly and directly (Tsilingiri and Rescigno, 2013). Therefore, the concept of postbiotics is based on the fact that the positive effects of the gut microbiota are mediated by the secretion of multiple metabolites (Zółkiewicz et al., 2020; Pothuraju et al., 2021; Vrzáčková et al., 2021). Notably, postbiotics are non-live bacterial products, and they do not include live microorganisms (Wegh et al., 2019; Zółkiewicz et al., 2020). Recently, it has been recognized that postbiotics may also significantly affect and strengthen gut microbiome, and they may be a new therapeutic option for cancer patients. Therefore, in this study, we described the possible role of postbiotics in the management of cancer patients. We focus on exopolysaccharides, cell wall fragments, tryptophan metabolites, enzymes, bacterial lysates, extracellular vesicles, and short-chain fatty acids (SCFAs) (Figure 1).

## 2. Exopolysaccharides

Microbes produce biopolymers that can be released outside the bacterial cell wall. These heterogeneous groups of substances are called exopolysaccharides (EPSs) (Zółkiewicz et al., 2020). Probiotic lactic acid bacteria produce EPSs, which provide anti-proliferative effects on various cancer cells. Notably, EPSs can modulate the development of tumors via multiple mechanisms regarding, among others, the promotion of apoptosis (Rahbar Saadat et al., 2019; Wu et al., 2021). In 2020, it was shown that EPSs isolated from *Rhizopus nigricans* (zygomycete filamentous fungus widely used in the pharmaceutical industry) induce colon cancer cell apoptosis both *in vitro* and *in vivo* through activating the AMP-activated protein kinase (AMPK) pathway (Lu et al., 2020). The impact of EPSs on apoptosis has been also confirmed in the study by Tahmourespor et al., with HT-29 colorectal cancer cell line (Tahmourespour et al., 2020). The authors reported that EPSs from *Pseudomonas aeruginosa* could induce apoptosis and necrosis (Tahmourespour et al., 2020). Similarly, Sun et al. investigated the effect of EPSs on the HT-29 cell line, but these EPSs were obtained from other bacteria, such as *Lactobacillus plantarum*-12, *L. plantarum*-14, *L. plantarum*-32, and *L. plantarum*-37 (Sun et al., 2021). It was observed that EPSs produced by *L. plantarum*-12 may inhibit the proliferation of the aforementioned cell line through the mitochondrial pathway (Sun et al., 2021). In another study, it was shown that the EPSs isolated from *Lactobacillus acidophilus* may upregulate the expression of tissue inhibitor of metalloproteinases-3 (TIMP-3), hypoxia-inducible factor-2α (HIF-2α), and hemeoxygenase-1 (HO-1) and downregulate the expression of vascular endothelial growth factor (VEGF) and hypoxia-inducible factor-1α (HIF-1α). Moreover, plasminogen activator inhibitor-1 (PAI-1) was increased. Therefore, these results suggest that EPSs derived from *L. acidophilus* have an antioxidative effect and inhibit the expression of genes that are involved in tumor angiogenesis (Deepak et al., 2016).

Notably, EPSs have an impact on not only colon cell lines but also hepatoma cells. HepG2 cells are non-tumorigenic cells, which present a high rate of proliferation (Donato et al., 2015). In the study by Yahya et al., it was noted that EPSs isolated from marine bacteria enhance antitumor activity in aforementioned cells by regulating the apoptotic gene and increasing the expression of toll-like receptors (TLRs) (Yahya et al., 2019). Recently, in 2021, it was shown that bacterial EPSs, such as EPS-6 and EPS-RS, induce apoptosis, and they may be promising agents in the treatment of hepatocellular carcinoma (Abdelnasser et al., 2021).

## 3. Cell wall fragments

The bacterial cell wall consisted of many components stimulating an immune response, e.g., gram-positive bacteria lipoteichoic acid (LTA) (Zółkiewicz et al., 2020), peptidoglycan (Wolf and Underhill, 2018), gram-negative bacteria lipopolysaccharide (LPS), lipoproteins, and mycobacterial lipoglycans (Ray et al., 2013). The potential antitumor effect of these components is not a new discovery. Even publications from the 80's described that fact. For example, the study by Usami et al. reported that LTA extracted from *Streptococcus* pyogenes inhibited the tumor growth of both solid- and ascites-type MethA fibrosarcoma, whereas other cell wall fragments, such as peptidoglycan, group-specific C-carbohydrate, or type-specific M protein, had no effect. Induction of tumor necrosis factor was depicted as the main mechanism by which LTA inhibited tumor growth (Usami et al., 1988).

Recent studies give us more complex insight into the interaction of cell wall fragments and cancer, indicating that a lot of molecules play a role in the process. The study by Arabzadeh et al. (2016) treated ovarian cancer cell line SKOV-3 with LTA, LPS, and recombinant human Il-6 alone or in combination with NF-kB inhibitor or STAT3 inhibitor. The treatment was applied in 4, 8, 12, 24, and 48 h. Then, the authors evaluated the expression of Wnt5A and ROR2 at the gene and protein levels. Wnt5a, a molecule participating in inflammatory processes, interacts with its receptor or co-receptor ROR2. It was indicated that the Wnt5A-ROR2 axis played a significant role in the migration and invasion of different types of cancers, influencing their polarity (Arabzadeh et al., 2016; Lyros et al., 2016; Sakamoto et al., 2017). Arabzadeh et al. proved that stimulation with LTA or Il-6 for 8 h induced relevantly increased expression of Wnt5A, whereas LPS, LTA, and Il-6 treatment led to a significant increase in ROR2 expression (after 48 h). In the presence of STAT3 or NF-kB inhibitors, Wnt5A expression was downregulated. In addition, LPS or LTA induction effect on ROR2 and Wnt5A expression was not present when STAT3 inhibitor was used, whereas both Il-6-induced ROR2 and Wnt5A expression levels were abrogated in the presence of STAT3 and NF-kB inhibitors. These results suggest that inflammatory mediators, such as LTA, LPS, or Il-6, can modulate ovarian cancer progression by changing inflammatory response and expression (Wnt5A-ROR2) patterns. Knowing that the blockage of ROR2 expression by using an anti-human antibody caused an 80% decrease in the migration of Wnt5A-transfected cells, it may be suspected that in the case of ovarian cancer, bacterial cell wall fragments may have anti-metastatic potential (Arabzadeh et al., 2016). Due to the limitations of the study caused by using only one cell line, the potential application of results in the treatment of ovarian cancer should be further investigated —first with a higher number of used cell lines and then in animal and clinically orientated studies. Another study conducted by Rezania et al. in prostate cancer showed that an individual cell line may not be enough to determine the effect of bacterial cell wall fragments on cancer. The analysis conducted with three cell lines, namely LNCaP, PC3, and DU145 (representing low, moderate, and high metastatic potential, respectively), treated with LPS and LTA at different concentrations and times showed that induction of immune response by these substances strongly depends on the expression profile of toll-like receptor, which is different in prostate cancer cell lines. Rezania et al. proved that the most clinically advanced cell lines had the most comprehensive TLR set (2014). Finally, it was revealed that LPS significantly induced increased proliferation of only DUI45 cells, whereas LTA stimulated the proliferation of all used prostate cancer cell lines. In addition, both LPS and LTA increased the adhesion capacity of the PC3 cell line, reducing the risk of metastasis. However, in LNCaP cells, LPS but not LTA treatment resulted in a relevant increase in invasion capacity (Rezania et al., 2014). Another study of prostate cancer cell line LNCaP conducted by Reader et al. investigated the effect of mycobacterial cell wall-DNA complex (MCC). They showed that MCC stimulated apoptosis of LNCaP cells and synthesis of Il-12 and GM-CSF by these prostate cancer cells (Reader et al., 2001). Moreover, the study by Hattar et al. in non-small lung cancer cell lines (adenocarcinoma A549 and squamous cell carcinoma H226) with a purified form of *Staphylococcus aureus* LTA showed a pro-proliferative effect on both used cell lines, whereas, in colorectal cancer HCT-116 cells, it was shown that miR-200c-3p expression was elevated after LPS treatment and had no anti-proliferative effect but inhibited migration, adhesion, and apoptosis (Hattar et al., 2017; Jiang et al., 2020). As it was mentioned above, LPS may both inhibit but also stimulate tumor growth. Several cell line studies indicate its role in carcinogenesis. As a component of *Helicobacter pylori*, it plays a role in gastric cancer, promoting proliferation and migration *via* an inflammatory mechanism (Li et al., 2019). Toll-like receptor 4 in cancer cells increases both cell survival and proliferation in hepatocellular carcinoma (Lipopolysaccharide-induced toll-like receptor 4 signaling in cancer cells promotes cell survival and proliferation in hepatocellular carcinoma - PubMed [WWW Document], 2013). Drawing a conclusion from mentioned studies, the effect of cell wall fragments on cancer may vary according to cell line stage, invasiveness, applied substance, and types of cancer. Future cell line studies should put emphasis on the wide diversity of used cell lines with a different invasiveness potential. The drawn conclusion cannot be treated as a general rule for all cancers, but the effect of each cell wall fragments, such as LPS or LTA, ought to be carefully investigated for each type of neoplasm, in addition to all pathological and clinical grades or stages. Only a rigorous analysis focused on the survival and other key clinical parameters may give an answer to the question: is it beneficial to use extracted and specific cell wall fragments in treatment? In addition to the cell line results, an animal study of colorectal cancer by Zhu et al. confirmed that LPS increases the excretion of VEGF-C through the TLR4-NF-κB/JNK pathways, promoting lymphangiogenesis, lymphatic metastasis, and pro-metastatic phenotype (Zhu et al., 2016).

On the other hand, fungal cell wall fragments are also an interesting part of the research. For example, yeast beta-D-glucan increased the release of tumor necrosis factor-alpha from murine macrophages, inhibited lipid peroxidation, and revealed antioxidant, antimutagenic, and antigenotoxic properties (Kogan et al., 2008). The study of the Lewis lung carcinoma cell line [LL/2] by Sadeghi et al. investigated the impact of beta-glucan (BG) on treatment-resistant lung cancer cells. It was proven that BG at concentrations of 800 and 100 μg/ml had meaningful cytotoxic effects on LL/2 cells, measured with the MTT test. In addition, RT-qPCR analysis revealed that Oct4 and Sox2 genes were downregulated in cells treated with BG, in comparison with the control. Knowing that these genes play a role in metastasis, the authors suggested the anti-metastatic potential of BG and the need for further studies confirming these results (Sadeghi et al., 2020). The study by Fortin et al. in colorectal cancer cell lines Hepa 1c1c7 and HT-29 confirmed the anti-proliferative effect of BG, indicating also that BG stimulated NAD(P): quinone reductase (QR) (Fortin et al., 2018a). The fact that QR is overexpressed in many cancers and plays a role in chemoresistance (Zhang et al., 2018) can be a limitation to its use in treatment. Although BG has anti-proliferative properties, it may also stimulate resistance to chemotherapeutic agents through QR. In addition, Fortin et al. emphasized that high amounts of glucan, high glucan/total sugar ratios, and low chitin/glucan ratios were associated with an increase in anti-proliferative and chemopreventive effects (Fortin et al., 2018a). A similar effect of BG on colon cancer prevention was observed in 1,2-dimethylhydrazine-treated rats (Fortin et al., 2018b). Another study by Fortin et al. reported that after administration of BG, QR activity increased, whereas reduction of aberrant crypt foci was proven, indicating anti-proliferative properties of BG (Fortin et al., 2018b).

**Figure 2:**
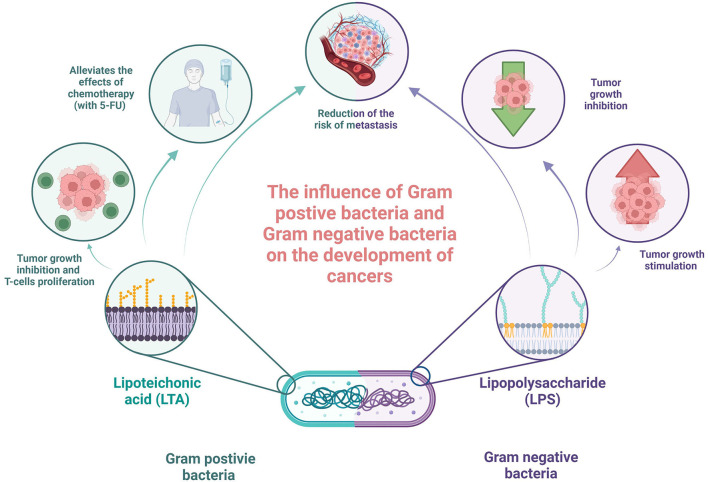
Summary of acting of cell wall fragments in the context of oncology. Own elaboration based on the literature (Xie et al., 2012; Ray et al., 2013; Guo et al., 2015; Wolf and Underhill, 2018). This figure was created using Biorender.com.

Although the results of studies describing immunomodulatory cell wall fragments are not fully clear and consistent, some studies tried to investigate the potential clinical applications of these molecules with common promising results. The study by Xie et al. investigated the combination of LTA extracted from *Bifidobacterium* with 5-fluorouracil (5-FU) on mice bearing inoculated hepatoma-22 cells, and it was shown that LTA enhanced inhibition of tumor growth, T lymphocyte proliferation, and IFN-gamma production by spleen cells, in comparison to 5-FU only (Xie et al., 2012). In the beginning, the high ratio of CD4(+), CD25(+), and regulatory T cell [CD4(+)CD25(+)T(reg)] in all spleen cells and elevated TIM-3 and Foxp3 proteins and mRNA expression levels were observed in the group of spleen cells of tumor-bearing mice. After treatment with LTA in combination with 5-FU, the CD4+CD25+Treg ratio degraded, whereas both Foxp3 and TIM-3 mRNA protein and mRNA expression levels were decreased. Although 5-FU has a similar effect alone, LTA significantly enhanced these properties (Xie et al., 2012). In addition, LTA alone or in combination with 5-FU increased the activity of NK cells and cytotoxic lymphocytes in the spleen (Xie et al., 2012). Knowing that Treg plays a role in resistance to immunotherapies and has immunosuppressive properties in cancer (Tanaka and Sakaguchi, 2017), the decrease in its level seems to be a promising result. In Tanaka and Sakaguchi's study (2017), increased cell-mediated immunity resembled by enhancing the activation of NK and cytotoxic lymphocytes may also stimulate anti-tumor immune response and should be further investigated. The study by Giuo et al. confirmed these results and added that LTA and 5-FU combination may also alleviate the side effects of chemotherapy (Guo et al., 2015). Another study by Cui et al. reported the efficiency of the combination of miR-27b-3p agomir and LTA in the treatment of gastric cancer. Reader et al. showed that both molecules have synergistic effects on gastric cancer treatment (2001). Moreover, it was proven that miR-27b-3p targets vascular endothelial growth factor C, inhibiting also the AKT pathway in AGS and N87 gastric cancer cells. It was also revealed that LTA inhibited proliferation, modulating the NF-κB pathway. In addition, the authors confirmed (using the established xenograft model of gastric cancer) that both miR-27b-3p agomir alone and LTA treatment inhibited tumor development, showing the most significant effect on the combination (Reader et al., 2001). On the other hand, Chakraborty et al. investigated the impact of LPS, LTA, and a combination of both on capsaicin efficiency in oral cancer. It was revealed that the presence of bacterial antigens interfered with capsaicin and reduced its efficiency in terms of the inhibition of cell proliferation and metabolism (the MT Glo assay) and increase in cell death (the Trypan blue exclusion assay and caspase 3/7 expression) (Chakraborty et al., 2021). These different effects of cell wall fragments on gastric and oral cancer cell lines again indicate the need for specific studies in each type of cancer separately. Last but not least, the mentioned study conducted by Caisová et al. applied the therapeutic mixture consisting of TLR agonist resiquimod, poly (I:C), and LTA in the mice model of melanoma. The obtained results seem to be promising because this combination caused the eradication of advanced stage progressive melanoma in 83% of mice and have significant anti-metastatic potential and relevantly reduced risk of tumor recurrence, indicating the role of innate and adaptive immunities in that process (Caisová et al., 2018). They also investigated the Panc02 murine model of invasive pancreatic tumor. However, the additional application of anti-CD40 was needed to obtain 80% response to treatment. A combination of only a basic therapeutic mixture resulted in a less efficient and not statistically significant response, influencing tumor growth, survival prolongation, and suppression of metastases (Caisová et al., 2018). The summary of the acting of cell wall fragments is presented in Figure 2.

## 4. Tryptophan metabolites

Amino acids contained in the human body play critical roles in not only nourishing but also preserving health and homeostasis. One of the 20 amino acids occurring naturally in the human body is tryptophan, which is indirectly responsible for body growth, maintaining emotional balance, stimulating the immune system, regulating the sleep schedule, and many others (Dell'Osso et al., 2016; Anaya et al., 2020; Martínez-Rodríguez et al., 2020). Since tryptophan (TRP) is an exogenic amino acid, it needs to be delivered with a daily diet. Pumpkin and sunflower seeds, sesame seeds, chia seeds, bananas, chocolate, eggs, cheese, beef, and soy products are high in this chemical, which is quickly absorbed from the digestive tract after intake (Davidson et al., 2022). After being absorbed from the digestive system, TRP undergoes many miscellaneous metabolic routes and is transformed into various chemical compounds, which are crucial in many different aspects of maintaining homeostasis (Anaya et al., 2020). Three vital metabolic pathways of TRP are the kynurenine pathway, serotonin pathway, and indole pathway. Understanding how the tryptophan-kynurenine pathway is controlled in different tissues, numerous biological functions of its metabolites have become an interdisciplinary subject. It is known that the kynurenine pathway is strongly regulated in the immune system, whereby in reaction to inflammation or infection, it induces immunosuppression (Cervenka et al., 2017). KP activation results in the formation of a variety of physiologically active metabolites such as kynurenine (KYN), kynurenic acid (KYNA), and quinolinic acid (QUIN). QUIN is one of the products of the kynurenine pathway and a precursor of nicotinamide adenine dinucleotide (NAD+), which is an important metabolite in TRP catabolism. According to the study by Moffett et al., in response to inflammation and infection, the level of QUIN increases which may lead to the enhancement or replenishment of cellular NAD+ levels. Even though the generation of NAD+ in immune system cells during health and illness is mostly unknown, it is believed that NAD+ requirements, in particular cell types, rise during inflammation, injury, and infection, and that this is required for proper immune cell response, which is vital in conducting efficient anticancerogenic processes (Minhas et al., 2019). Another TRP metabolite of great importance, such as kynurenic acid (KYNA), was proven to have an inhibitory effect on the proliferation of various cancer cell lines such as colon cancer, renal cancer, and glioblastoma cells. In the study by Walczak et al., researchers came to the conclusion that KYNA has an inhibitory effect on the activation of PI3K/Akt and MAPK signaling pathways in colon adenocarcinoma HT-29 cells (Walczak et al., 2014). Additionally, indoleamine 2,3-dioxygenase (IDO) and its metabolite produced from TRP, kynurenine (KYN), are recognized as the most immunosuppressive mediators with proven effectiveness in the development of cancer as it may stimulate apoptosis in cells with higher levels of Caspase-3 and Caspase-9 activities and through encouraging *de novo* NAD+ production, it can defend against oxidative stress which may be cancerogenic (Abd El-Fattah, 2022).

TRP can be transformed into serotonin (5-hydroxytryptamine, 5-HT) *via* a distinct metabolic process known as the serotonin pathway, due to an enzyme called tryptophan hydroxylase (TPH). According to several types of scientific research, serotonin, which is widely recognized for its effects on the human body as a neurotransmitter and a motility mediator in the gastrointestinal tract, may also have a significant impact on carcinogenesis (Balakrishna et al., 2021). Researchers suggest that the impact of serotonin on cancer cells may differ depending on its concentration in said cells. On the one hand, a high amount of serotonin presents a stimulatory effect on the growth of aggressive cancer cells via engaging with 5-HT1 and 5-HT2 receptors, but on the other hand, serotonin, when given in low doses, would reduce the blood flow which would obstruct tumor growth. According to the study conducted by Sonier et al., serotonin stimulates the development of malignant breast cells in part via 5-HT2A receptors. RT-PCR, Western blotting, and immunofluorescence analyses were used to assess the expression of the 5-HT(2A) serotoninergic receptor subtype in MCF-7 cells. Researchers concluded that serotonin and the selective 5-HT2A receptor agonist enhanced cell proliferation in a concentration-dependent manner in the human breast cancer cell line MCF-7 (Sonier et al., 2006). In another interesting research demonstrated by Pai et al. (2009) it was brought to attention that during tumor progression, the expression of the enzyme responsible for the transformation of TRP into serotonin was increased, which suggested that higher levels of serotonin favor tumor progression. When it comes to the indole pathway, unabsorbed Trp in the intestines is subsequently converted by the microbiota into indole and indolic chemicals, such as indole-3-pyruvate, indole-3-acetamide, indole-3-acetaldehyde, indole-3-acetic acid, indole-3-lactic acid, and indole-3-propionic acid. These indolic chemicals are capable of binding with aryl hydrocarbon receptors (AhRs), which leads to the improvement of intestinal homeostasis but also improves barrier function and tight junctions. All that proves a significant influence on host health due to Trp metabolism by bacteria (Chimerel et al., 2014; Gao et al., 2018).

With the exception of indoxyl sulfate, a uremic toxin implicated in chronic renal and vascular illnesses (Lin et al., 2012), and the bulk of indoles and indole derivatives has been shown to protect against CRC. It was also proven that indolic compounds may promote pro-inflammatory and anti-inflammatory effects since they can act as AhR ligands (Alexeev et al., 2018). Anti-inflammatory effects are provided by the fact that indoles manage to suppress the expression of pro-inflammatory signals such as IL-8 and NF- kB while increasing the expression of anti-inflammatory cytokines such as IL-10 (Hendrikx and Schnabl, 2019). Even though indolic compounds have a generally positive impact on maintaining homeostasis, the study shows that in later stages of cancer, the production of IL-22 stimulated by said compounds may promote the progression of tumor (Hernandez et al., 2018; Busbee et al., 2020).

## 5. Extracellular vesicles

The intestinal microbiota can produce a bilayer named extracellular vesicles (EVs) (González-Lozano et al., 2022). EVs are membrane-derived lipid bilayers surrounded by cytosolic compounds, microbial cells, proteins, nucleic acids, and pro-inflammatory toxins (e.g., polysaccharides) (Kim et al., 2015). Depending on bacteria—EVs released by gram-negative bacteria differ in size and the structure of the membrane—the vesicles produced by gram-negative bacteria are referred to as outer membrane vesicles (OMVs), which are generally larger (20–200 nm in diameter) and more complicated in membrane structure (Kim et al., 2015).

*Lactobacillus* spp. are examples of bacteria forming MVs with properties relevant to the tumorigenic process. In an animal model of colorectal cancer, a number of *Lactobacillus* species—*L. casei, L. rhamnosus* GG, and *L. acidophilus*—have documented anti-cancer effects probably through the phenomena of extracellular vesicles (Goldin et al., 1996; McIntosh et al., 1999). One potential mechanism may be related to the transmission of information—DNA, LPS, and proteins contained in MVs.

It has been demonstrated that the presence of MVs may improve the response to the appearance of tumor cells by regulating the process of cell differentiation and apoptosis (Rafter, 2004). It appears that extracellular vesicles produced by gram-positive bacteria, such as *L. rhamnosus* GG (LrGG-MV), may have potential anti-cancer activities (González-Lozano et al., 2022). It has been tentatively confirmed by preliminary results from the observations of cellular models of colorectal cancer, where MVs have shown anti-proliferative effects (through probable modulation of carcinoembryonic antigen (CEA) gene expression) (Keyhani et al., 2022). Another cell model - liver cancer tumor (Hep G2) membrane vesicles inhibited cell proliferation via bax/bcl-2 (Behzadi et al., 2017).

## 6. Short-chain fatty acids

Anti-cancer effects are also shown by the best-known postbiotics which are short-chain fatty acids (SCFAs) (Kazmierczak-Siedlecka et al., 2022a). Notably, the SCFA pool (measured in stool samples) is changed in CRC patients, which has been recently shown in 2023 (Kazmierczak-Siedlecka et al., 2023). This study included 15 colorectal cancer patients in the preoperative period. The proportion between SCFAs (which should be 3:1:1 for acetate, propionate, and butyrate, respectively) was altered (especially observed as the low concentration of butyrate) (Kazmierczak-Siedlecka et al., 2023).

It seems that SCFAs, especially butyric acid, may be involved in anti-cancer effects by altering the cellular response to metabolic and oxidative stress (Vrzáčková et al., 2021; Kazmierczak-Siedlecka et al., 2022a). The best-studied butyric acid may have anti-proliferative, antimutagenic, and anti-inflammatory effects via the regulation of nuclear factor erythroid 2-related factor (Nrf-2) and Kelch-like ECH-associated protein 1 (Keap1) as well as the activation of 5′ AMP-activated protein kinase (AMPK) (Vrzáčková et al., 2021). The “butyrate paradox” observed in cancer-lesioned intestinal cells involves a different transport, utilizing butyric acid (Charney et al., 1998; Gonçalves and Martel, 2013). In non-tumor-lesioned intestinal epithelial cells, butyric acid is transported via transponders located on the apical side of colonocytes—monocarboxylate transporter 1 (MCT1), sodium-coupled monocarboxylate transporter 1 (SMCT1), breast cancer resistance protein (BCRP), and the SCFA/HCO3 exchange (Cuff et al., 2005; Thibault et al., 2007). Unfortunately, the transportation of butyrate via SMCT1, MCT1, or BCRP is significantly downregulated in many types of cancers—the only way is to provide butyrate in exchange for HCO3 (Vrzáčková et al., 2021). As a result of tumor transformation, relevant metabolic changes occur, and the cell switches to glucose metabolism (converted to lactate). Under these conditions, other glucose energy metabolites (including butyrate) are not exploited for energy production (via beta-oxidation), and butyric acid accumulates in the cytoplasm and then migrates to the nucleus, where it acts as an inhibitor of histone deacetylases (HDACs). As a result, histones are “loosened” by acetylation that allows access for transcription factors to DNA which leads to the expression of various proteins and induces oxidative stress and ultimately apoptosis (Donohoe et al., 2011; Burgess, 2012).

The anti-cancer effect of butyrate may be linked to its antioxidant role. Its addition to cultures of human colonocytes and colon cancer cells (HT29 and HT29 19A) mitigated the destructive effects of free radicals (Rosignoli et al., 2001). Interestingly, butyrate acted as an activator of the induction of certain antioxidant enzymes (catalase and metallothionein) and induced an increase in glutathione-S-transferase levels in colorectal adenocarcinoma cells and other types of cancer cells (Ebert et al., 2003).

*In vitro* studies showed that butyrate

- Stimulated the autophagy by the elevation activity cascade of kinases [B1/AMP-activated protein kinase (LKB1/AMPK)] in colorectal carcinoma (Zhang et al., 2016; Luo et al., 2019);
- Mitigated tumor invasion by activity and level of metalloproteinases, such as MMP-2 and MMP-9 (Vrzáčková et al., 2021);
- Activated chemotaxis and programmed cell death (Mirzaei et al., 2021).

An extremely interesting postbiotic seems to be tributyrin—a triacylglycerol containing three molecules of butyrate that exhibits chemopreventive properties at millimolar levels. In opposite to butyrate, tributyrin has a longer half-life, however, induces apoptosis and modulates histone acetylation, similar to butyrate (Watkins et al., 1999; Maier et al., 2000). There are scientific studies that may point to a negative role of SCFA in the tumorigenesis process. The study by Matsushita et al. (2021) showed that in prostate-specific Pten-knockout mice, SCFA supplementation promoted tumor growth by increasing IGF1 levels and activated local MAPK and PI3K signaling in the tumor-transformed prostate. In another study (Xie et al., 2021), rats were given 150 mM acetate, propionate, or butyrate in drinking water for 4 weeks. After that, expressions of the intestinal barrier proteins, such as P-glycoprotein (P-gp) and breast cancer resistance protein (BCRP), were recorded. Both proteins are key ATP-binding cassette (ABC) efflux transporters involved in the construction of the intestinal barrier. It was noticed that the expression of intestinal P-gp was decreased among rats, while the expression of intestinal BCRP was increased, especially in the group with butyric acid (followed by propionate and acetate).

Fermentation of fiber into SCFAs is a factor in reducing the incidence of colorectal cancers. Effects appear to include changes in fecal pH and acceleration of gut transit time. Such a phenomenon was confirmed in a study on health volunteers consuming wheat bran, observing an increase in butyrate content in stool and faster intestinal transit (Lewis and Heaton, 1997). Interesting results were obtained in the observation of 29 volunteers (obese or overweight) with colon cancer. The volunteers consumed an additional portion of heat-stabilized rice bran (30 g/day) and navy beans (35 g/day) for 28 days. The study hypothesized that the products of their fermentation by the gut microbiota may affect gut health. Dietary interventions using these foods should be studied to modulate colon cancer risk. Studies have shown that the consumption of rice bran led to an increase in stool SCFAs (propionate and acetate), after just 14 days of fiber supplementation (Sheflin et al., 2017). Ubachs et al. (2021) have shown how the content of SCFAs in the stool of 107 patients with cancer cachexia (during pancreatic cancer, lung cancer, and breast cancer as well as ovarian cancer in humans) is different in comparison with healthy controls (household partners, *n* = 76). Among cachectic patients, fecal concentrations of all SCFAs were lower than in the control (especially acetate). Ubachs et al. (2021) conclude that their results should open the way for further research into the role of gut microbiota metabolites in cancer cachexia and its potential use as a therapeutic target.

## 7. Conclusion

We concluded that discussed metabolites secreted by microbes may have direct as well as indirect positive impacts on gut microbiota, maintaining homeostasis. Postbiotics, depending on their build and origin, contribute to various biological and chemical processes in the human body by altering the progression of cancerous cells. Studies show that exopolysaccharides extracted from specific bacteria not only have anti-proliferative effects on cancer cells but also have an impact on mechanisms, which induce apoptosis of cells and even contribute to the inhibition of tumor angiogenesis. Second, bacterial cell wall fragments are well known for inducing a response in the immune system. According to many different studies, these components, when extracted and used in a specific way, may intervene in processes that are vital in carcinogenesis such as cancer cell proliferation, modifying the ability of those cells to infiltrate other tissues and stimulating tumor necrosis factor. Different groups of chemical compounds that are of great importance are metabolites of TRP. Products of the kynurenine, serotonin, and indole pathways were proven to have a great impact on inhibiting the progression of cancer as they contribute to enabling proper immune response and have indirect anti-proliferative effects on various cancer cell lines by reducing the blood flow to the tumor or modulating specific signaling pathways. Another postbiotics brought to attention are extracellular membrane vesicles that can regulate cell differentiation and apoptosis, contributing to inhibiting the development of cancer. The impact of anti-cancer was also documented in studies dedicated to the most recognizable postbiotics, i.e., SCFAs. These compounds have an influence on how cells respond to metabolic and oxidative stress and may manifest the influence on antimutagenic, anti-inflammatory, and anti-proliferative processes as they are vital in maintaining homeostasis. Taking everything into consideration, we believe that alterations caused by postbiotics may induce positive effects throughout the anti-cancer therapy process in cancer patients and therefore might be considered a new supportive therapeutic method of treatment.

## 8. Clinical protocols using or stimulating the production of postbiotics in patients

PS was essentially produced by *L. acidophilus* (10307) obtained from Microbial Type Culture Collection, India. Essentially, EPS was produced in an optimized medium (3% sucrose, 1% yeast extract, 0.2% K_2_HPO_4_, 0.2% MgSO_4_, and 2% NaCl) and extracted by the cold ethanol method. Essentially, the method involves the treatment of the cell-free supernatant with three volumes of ice-cold ethanol and incubated for 2 h. The precipitate was collected, dissolved in water, dialyzed, and purified by anion exchange DEAE methacrylate column chromatography, eluted with NaCl gradient. The extracts containing EPS were pooled, and the EPS was dried using a vacuum dessicator (Wang et al., 2014).

## Author contributions

AK, KK-S, and ES contributed to the conception and design of the study. AK and KK-S wrote the first draft of the manuscript. BS, DM, JP, LC, and LM wrote the sections of the manuscript. FR, LK, and ES participated in the study design and coordination and reviewed the final draft. All authors contributed to the manuscript revision, read, and approved the submitted version.
