# Supplementary material for: Postbiotics in oncology: science or science fiction?
Source: Front Microbiol. 2023 Aug 7;14:1182547. doi: 10.3389/fmicb.2023.1182547 (PMC10440707; doi:10.3389/fmicb.2023.1182547)
Supplement: Supplementary file 1 [file Table_1.DOCX]

| Postbiotic | Action | Model  (*in vitro* or *in vivo*) | Source |
| --- | --- | --- | --- |
| **Cell line** | | | |
| Exopolysaccharides isolated from *Pseudomonas aeruginosa* | Induction apoptosis | HT-29 colorectal cancer cell line | Tahmourespour et al., 2020 |
| Exopolysaccharides isolated from *Lactobacillus plantarum*-12, *L. plantarum*-14, *L. plantarum*-32, *L. plantarum*-37 | Induction apoptosis | HT-29 colorectal cell line | Sun et al., 2021 |
| Exopolysaccharides isolated from *Lactobacillus acidophilus* | Antioxidative effect and inhibiton the expression of genes involved in tumour angiogenesis and survival. | HCT15 colon adenocarcinoma cell  and CaCo2 cell line | Deepak et al., 2016 |
| Exopolysaccharides isolated from marine bacteria *Brevundimonas subvibrioides* MSA1, *Bacillus thuringiensis* E4, *Bacillus amyloliquefaciens* MGA2, *Pseudomonas fluorescens* SGA3, and *Advenella Kashmirensis* NRC-7. | Induction apoptosis | HepG2 cells | Yahya et al., 2019 |
| Exopolysaccharide EPS-6 and EPS-RS from Bacillus megaterium SAmt17 (KP733903) and Bacillus subtilis Subsp. Subtilis SAmt3 (KP733900) | Induction apoptosis | HepG2 cells | Abdelnasser et al., 2021 |
| Lipoteichoic acid and Lipopolysaccharide A | Strong downregulation of Wnt5A expression (protein family, is implicated in inflammatory processes) | Ovarian cancer cell line SKOV-3 | Arabzadeh et al., 2016 |
| Lipoteichoic acid and Lipopolysaccharide A | Induction and increase proliferation DUI45 cells by LPS.  Induction apoptosis by LTA.  LPS increase of invasion capacity | Prostate cancer lines LNCaP, PC3 and DU145 | Rezania et al., 2014 |
| Mycobacterial cell wall-DNA complex (MCC) | Stimulation of apoptosis and synthesis of Il-12 and GM-CSF | Prostate cancer cell line LNCaP | Reader et al., 2001 |
| LTA purified from *Staphylococcus aureus* | Pro-proliferative effect | Non-small-cell lung cancer cell lines -adenocarcinoma A549 and squamous cell carcinoma H226 | Hattar et al., 2017; |
| Lipopolysaccharide S | Inhibition of migration, adhesion and apoptosis | Colorectal cancer HCT-116, colorectal carcinoma cell lines HT-29 and SW480 | Jiang et al., 2020 |
| Fungal beta-D-glucan from *Saccharomyces serevisiae* | Antioxidant, antimutagenic and antigenotoxic properties | Murine macrophages | Kogan et al., 2008 |
| Extracts from cell wall from *Saccharomyces boulardii* and *Kluyveromyces marxianus* | Anti-proliferative and chemopreventive effect | Colorectal cancer cell lines Hepa 1c1c7 and HT-29 | Fortin et al., 2018a |
| Fungal of beta- glucan from *Candida albicans* | Anti- metastatic effect | Lewis lung carcinoma cell line (LL/2) | Sadeghi et al., 2020 |
| Mycobacterial cell wall-DNA complex (MCC) | Induction apoptosis, escalations the synthesis of IL-12 and GM-CSF | LNCaP cells  androgen-sensitive human prostate adenocarcinoma cells | Reader et al., 2001 |
| Lipopolysaccharide S, Lipopolysaccharide A and Lipoteichoic acid (from Escherichia coli O111:B4,  LTA (from *Streptococcus pyogenes,* with  capsaicin | Inhibition oral cancer cell proliferation and metabolism (and increases cell death | Oral cancer cells Cal 27 | Chakraborty et al., 2021 |
| Tissue culture media, media supplements, laminarin from Laminaria digitata, mannan from Saccharomyces cerevisiae, GM-CSF, TNF-α, lipoteichoic acid (LTA) from Bacillus subtilis, | Eradication of advanced stage progressive melanoma in 83% of mice, acquisition of resistance to tumor re-transplantation, and potential anti-metastatic effect | Melanoma in murine B16-F10 model | Caisová et al., 2018 |
| Kynurenic acid (KYNA) | Potential chemopreventive agent in colon cancer or supportive agent in standard cancer chemotherapy | HT-29 cells | Walczak, K., at al. 2014 |
| Serotonin and the selective 5-HT2A receptor agonist | Enhancement cell proliferation | Human breast cancer cell line MCF-7 | Sonier et al., 2006 |
| Serotonin | Tumour progression | Human breast cancer cells (MCF7, MDA-MB-231 and T47D | Pai et al., 2009 |
| Bacterial extracellular vesicles (EVs) *Lactobacillus rhamnosus GG* (LDEVs) | Increase cell apoptosis. | Liver cancer tumor (Hep G2) | Behzadi et al., 2017 |
| Butyric acid | Induction cell-cycle arrest and differentiation | HT-29 and HCT116 cells | Cuff et al., 2005 |
| Butyric acid | Mitigation the destructive effects of free radicals | Colon cancer cells (HT29 and HT29 19A | Rosignoli et al., 2001 |
| Butyric acid | Chemoprotection by increasing detoxification capabilities in the colon mucosa | Colon cancer cell line HT29 | Ebert et al., 2003 |
| Butyric acid | Stimulation the autophagy | Colorectal carcinoma cells HCT-116 and HT-29 | Luo et al., 2019 |
| Butyric acid | Suppression colorectal cancer cell proliferation, induction autophagy | Human colorectal cancer cell lines (HCT-116 and HT-29) | Zhang et al., 2016 |
| Butyric acid and tributyrin | Act as potent apoptotic agents | Hep G2 cell | Watkins et al., 1999 |
| Tributyrin | Act as potent apoptotic agents | Human prostate cancer cell lines LNCaP, PC-3 and TSU-PR1 | Maier et al., 2000 |
| **Mixed cell line and animal model** | | | |
| Short chain fatty acids SCFA | Promotion of proliferation of prostate cancer cells. | Prostate-specific Pten knockout mice  and cancer cell lines DU145 and 22Rv1 | Matsushita et al., 2021 |
| Lipopolysaccharide from *Escherichia coli 055:B5* | Enhancement cancer cell motility and promotion human dermal lymphatic endothelial cells’ capacity of tube-like formation  Acceleration lymphangiogenesis and lymph node metastasis in mice. | Colorectal cancer cell lines (sw480, HCT116)    Nude mice. | Zhu et al., 2016 |
| Lipopolysaccharide S from Helicobacter pylori | Proliferation and migration of tumor cells via CXC chemokine receptor 7 (CXCR7) | Gastric cancer cell lines (SGC7901, AGS, MGC-803, MKN-45 and BGC823 and  nude mice (BALB/C-nu/nu | Li et al., 2019 |
| Exopolysaccharides (EPSs) isolated from *Rhizopus nigricans* | Induction apoptosis | Colon cancer cell CT26 and male BALB/c mice | Lu et al., 2020 |
| **Animal model** | | | |
| Extracellular vesicles from *Lactobacillus casei subsp. rhamnosus strain GG* | Before and during carcinogen treatment - decrease in the incidence of colon tumors and the number of small intestinal and colon tumors per tumor-bearing animal | Male Fischer rats | Goldin et al., 1996 |
| Extracellular vesicles from  freeze-dried bacteria *Lactobacillus acidophilus* (Delvo Pro LA-1), *Lactobacillus rhamnosus* (GG), *Bifidobacterium animalis* (CSCC1941), and *Streptococcus thermophilus (*DD145) | Inhibition of intestinal tumors | Male Sprague-Dawley rats zine (DMH) | González-Lozano et al., 2022 |
| Butyrate (secrecion induced by indole-3-carbinol (I3C) | Reduction colonic inflammation, protection of the mucus layer, | Female BALB/cJ and C57BL/6 | Busbee et al., 2020 |
| Lipoteichoic acid (LTA from Bifidobacterium with combination with 5-Fluorouracil (5 FU) | Inhibition the proliferation of tumor and induced obvious apoptosis | Tumor-bearing mice  Balb/c | Guo et al., 2015 |
| Lipoteichoic acid (from Bifidobacterium with combination with 5-Fluorouracil (5 FU) | Aggravations inhibition of tumour growth, T lymphocyte proliferation and IFN- gamma production by spleen cells.  LTA alone or in combination with 5-FU increased activity of NK cells and cytotoxic lymphocytes in spleen | Mice bearing inoculated hepatoma-22 cells | Xie et al., 2012 |
| Cell wall extracts from *Saccharomyces boulardii* | Colon cancer prevention by reduction acytivity quinone reductase (QR) and fecal enzymes (β-glucosidase and β-glucuronidase) | Male F344 Rats | Fortin et al., 2018b |
| Lipoteichoic acid (LTA) from *Streptococcus pyogenes* | Induction of tumour necrosis | BALB/c mice | Usami et al., 1988 |
